# USING THE INTERPRETIVE FRAMEWORK TO CREATE AND DELIVER MEANINGFUL GROUP ACTIVITIES FOR OLDER ADULTS

**DOI:** 10.1093/geroni/igae098.3509

**Published:** 2024-12-31

**Authors:** Anna Tulloh, Megan Westmore, Keith Anderson, Rebecca Mauldin

**Affiliations:** University of Mississippi, Oxford, Mississippi, United States; The University of Texas Arlington, Arlington, Texas, United States; University of Mississippi, Oxford, Mississippi, United States; University of Texas at Arlington, Arlington, Texas, United States

## Abstract

Activity programming is recognized as a vital component in supporting engagement and improving quality of life for older adults. Despite the ubiquity of such programs and a plethora of research exploring both why meaningful activities are important for older adults and what makes an activity meaningful, current guidelines for activity development fail to offer a standardized framework. Without a clear framework for developing meaningful group activities, a potential for inconsistencies in activity appeal, quality, and effectiveness exists. This paper suggests an innovative solution by drawing on the field of interpretation—a discipline traditionally associated with environmental, historical, and cultural education. Interpretation provides principles and practical guidance for creating engaging, relevant, enjoyable, and personalized experiences for a wide range of audiences. In this paper, we present a brief history of interpretation and describe its principles, applications, and the “interpretative equation.” Using data from field notes collected during a study of an online nature-based group activity in an assisted living community, we describe how the program was developed, implemented and adapted in alignment with the elements of the interpretive equation. This equation combines activity-related knowledge (e.g. gaining a deeper understanding of the environment presented online), an understanding of participants (e.g. increasing knowledge of individual’s dementia-related support needs), and appropriate techniques (e.g., incorporating reminiscence) to achieve an interpretive “opportunity” (i.e., a meaningful activity in our formulation). Our results demonstrate how interpretation can be a powerful tool in bridging the gap between the theory and practice of meaningful activity development.

